# Understanding Oxidative Stress in *Aedes* during Chikungunya and Dengue Virus Infections Using Integromics Analysis

**DOI:** 10.3390/v10060314

**Published:** 2018-06-09

**Authors:** Jatin Shrinet, Neel Sarovar Bhavesh, Sujatha Sunil

**Affiliations:** 1 Vector Borne Diseases, International Centre for Genetic Engineering and Biotechnology (ICGEB), Aruna Asaf Ali Marg, New Delhi 110067, India; jatbioinfo@gmail.com; 2 Transcriptional Regulation, International Centre for Genetic Engineering and Biotechnology (ICGEB), Aruna Asaf Ali Marg, New Delhi 110067, India

**Keywords:** *Aedes*, Arbovirus, chikungunya, dengue, proteomics, transcriptomics, metabolomics, oxidative stress

## Abstract

Arboviral infection causes dysregulation of cascade of events involving numerous biomolecules affecting fitness of mosquito to combat virus. In response of the viral infection mosquito’s defense mechanism get initiated. Oxidative stress is among the first host responses triggered by the vector. Significant number of information is available showing changes in the transcripts and/or proteins upon Chikungunya virus and Dengue virus mono-infections and as co-infections. In the present study, we collected different -omics data available in the public database along with the data generated in our laboratory related to mono-infections or co-infections of these viruses. We analyzed the data and classified them into their respective pathways to study the role of oxidative stress in combating arboviral infection in *Aedes* mosquito. The analysis revealed that the oxidative stress related pathways functions in harmonized manner.

## 1. Introduction

Being an anautogenous species, *Aedes* mosquito depends on blood feeding for providing nutrients for its maturing eggs [1,2]. The process of blood feeding, however, has its toll on the mosquito, resulting in induction of immense oxidative stress [3]. The scenario becomes further complicated when the blood that is ingested by the mosquitoes is infected with pathogens that may result in a fitness cost to the mosquito [4].

Arboviruses are a class of RNA viruses that are transmitted by the mosquitoes. Among these, most important are the dengue virus (DENV) and chikungunya virus (CHIKV) owing to their pathogenicity in humans, thereby being a major public health issue across the globe [5]. Both these viruses are transmitted by the *Aedes* species and are known to co-infect the same mosquito [6]. DENV is a member of the Flavivirus and is a single-stranded positive-strand virus with an RNA genome of approximately 11 kb length. CHIKV is an Alphavirus and is also a single-stranded positive RNA virus, approximately 11 kb in length [7,8]. In spite of these similarities, the viruses differ from each other in terms of their genome composition and replication mechanisms [9,10,11,12]. Upon arboviral infection, a cascade of events involving numerous biomolecules affect the mosquito that either results in combating the pathogen or allowing the pathogen to reside within the vector. Irrespective of the final outcome, invasion of the pathogen initiates the mosquito defense mechanism and the induction of oxidative stress is among the first host responses that are triggered by the vector. Much information is available with respect to alterations in the transcripts and/or proteins during the infection of these viruses both as monoinfections and as coinfections.

With the advent of new techniques for studying and understanding the complexity of biological processes, the amount of data generated during the processes is also increasing [13]. These advancements in different areas of biology have led to the rise of the -omics era [14]. Previous studies focused mostly on single omics, but every biomolecule such as mRNA, proteins, and metabolites are interconnected to each other and they may or may not work in harmony [13]. For example, a gene may transcribe into an mRNA but due to post-transcriptional modification it may or may not get translated into the protein(s). To understand this complex mechanism and for gathering some important information, studies should be performed not just on one but on different aspects of the cellular processes and they should be integrated to present a clear picture [15].

The present study was initiated to understand oxidative stress in the mosquito upon arboviral infections. Using proteomics, transcriptomics, and metabolomics data available in the public database and data generated in our laboratory related to CHIKV, DENV, or co-infections of these viruses, we analyzed the role of oxidative stress in combating arboviral infection in *Aedes* mosquito.

## 2. Materials and Methods

### 2.1. Mosquitoes Rearing and Virus Infection

Mosquitoes rearing and nano-injections were performed using protocol described in our earlier study [16]. Briefly, Indian strain of *Aedes aegypti* collected from New Delhi region and adapted to our laboratory conditions were kept at 28 ± 1 °C temperature and 75–80% humidity. The female mosquitoes used in the study were from F9 generation. Sterile sucrose solution was prepared and adults were fed on this solution. Female mosquitoes that were 4–6 days old were kept on ice and four groups of 100 mosquitoes each were formed and injected with either PBS or viruses in the thoracic region. The group injected with PBS served as control. Approximately 10^6^ pfu/mL of CHIKV and DENV were injected separately into 100 mosquitoes each; equal quantities of both the viruses were mixed and 10^6^ pfu/mL of this mixture was injected into the last group of 100 mosquitoes. Mosquitoes were allowed to recover. After 24 h post injection [16], whole legs were removed from its base at the thoracic region and the hemolymph was directly collected as droplets. NMR buffer (Total volume 500 mL: 380 mL H_2_O; 0.4 gram DSS; 10.05 gram Na_2_HPO_4_·7H_2_O; 5 mL NaN_3_ (4%); 100 mL D_2_O; pH 7.4) was added to the hemolymph and processed for the NMR experiments. Experiments were performed three times for each of the groups: namely, mock-injected mosquitoes (MM), CHIKV-infected mosquitoes (CM), DENV-infected mosquitoes (DM), and mosquitoes co-infected with both the viruses (CD).

For checking the status of the mono- and co-infections in individual nano-injected mosquitoes, five mosquitoes from each condition were taken at 24 h.p.i., 48 h.p.i. and 72 h.p.i. Viral RNA was extracted from whole mosquitoes separately and qRT-PCR was performed to quantify viral RNA copy number. In case of quantification of viral RNA copies in hemolymph, the hemolymph from the pool of 50 mosquitoes at 24 h.p.i. was collected in trizol and qRT-PCR was done to confirm the presence of CHIKV and DENV in *Aedes aegypti*.

### 2.2. NMR Spectroscopy

NMR spectroscopy and data analysis were performed using the steps mentioned elsewhere [17]. Briefly, all hemolymph samples dissolved in NMR buffer were used for 1D ^1^H NMR spectroscopy. The final volume of the sample was 200 µL. The NMR experiments were performed at 298 K on a Bruker Avance III Spectrometer equipped with cryogenic triple-resonance TCI probe, operating at the proton field strength of 500 MHz. The Carr-Purcell-Meiboom-Gill (CPMG) echo sequence was used to partially suppress protein signals. A total CPMG delay of 300 ms was used with an echo time of 200 ms. All one-dimensional ^1^H NMR spectra were measured with 32 scans with a relaxation delay of 4 s. For each sample free induction decays (FIDs) were collected with a spectral width of 10,000 Hz and acquisition time of 3.28 s (*t*_1max_). The peaks were processed using Topspin2.1 (Bruker AG, Falländen, Switzerland). Base line correction, peak alignment, and scaling of peaks were performed and referenced to DSS at 0 ppm. Peaks in the 6.0–4.5 ppm region were excluded to remove water signals.

### 2.3. NMR Data Analysis

Peaks were picked for each sample separately and were further analyzed. Owing to the high dimensionality and the complexity of the NMR data, multivariate statistical analysis of the data is required. All the statistical analysis and pathway annotations were carried out using MetaboAnalyst web tool (www.metaboanalyst.ca). MetaboAnalyst accepts different types of data format generated either from NMR or MS experiments. The tool performed data integrity check. Missing values were imputed using Singular Value Decomposition (SVDIMPUTE) algorithm. Row-wise data were normalized using sample median, log transformation was performed for normalization of the data, and autoscaling algorithm was used for data scaling. For multi-group analysis, one-way ANOVA was performed, followed by post-hoc analyses using Tukey’s HSD (honest significant difference) test. For identifying important features in pair-wise data analysis, three tests were performed: namely, fold-change analysis with threshold value of 2, *t*-test, and volcano plot (which is a combination of the first two). For predicting variance in the samples, multivariate analysis like principal component analysis (PCA) and partial least squares discriminant analysis (PLS-DA) were performed. The NMR peaks found to be significantly modulated were annotated using COLMAR web tool. These servers perform the search for the peaks in Madison-Qingdao Metabolomics Consortium Database (MMCD) (mmcd.nmrfam.wisc.edu) and annotate the peaks by identifying the corresponding metabolites. The significant pathways involved in the infections were also identified using MetaboAnalyst tool.

### 2.4. -Omics Data Collection

Apart from in-house generated transcriptomics, proteomics, and metabolomics data related to CHIKV, DENV mono- and co-infection, all other data from the previous studies were searched and downloaded from public databases. To obtain the already published material, searches were performed in databases like PubMed and also using other search engines, with a combination of key words like “Aedes” OR “mosquito” AND “transcriptomics” OR “proteomics” OR “metabolomics” OR “co-infection” AND “chikungunya” OR “dengue” or both. Studies not performed using high-throughput omics approach were excluded.

### 2.5. Integrated Pathway Annotation

The selected proteomics and transcriptomics studies were screened and all the data were divided into different groups such as salivary glands, midgut, carcass, and whole body. After classification, the proteins and transcript IDs of differentially regulated biomolecules from different studies were collected and converted into UniProt IDs and Entrez gene IDs using UniProt ID mapping web tool and VectorBase, respectively [18]. This step was done to maintain homogeneity in IDs during the analysis and to reduce bias in analysis. *Aedes albopictus* IDs were also converted into *A. aegypti* IDs by identifying the suitable homologs using Biomart tool of VectorBase. Pathway analysis was performed using KOBAS 3.0 web server [19]. Pathways with *p* ≤ 0.05 were considered significant. Details of the workflow followed in the study are shown in Supplementary Figure S1.

## 3. Results

### 3.1. Infection of CHIKV and DENV in Mosquitoes

**Figure 1:**
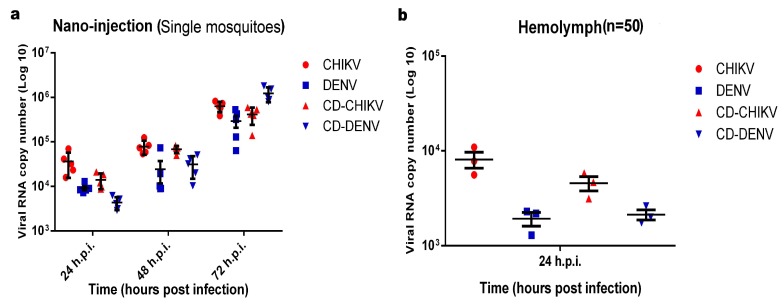
The figure shows quantification of viral RNA copies scaled to log 10. (**a**) Individual mosquitoes were checked for the presence of CHIKV and DENV RNA copies in both mono-infection and co-infection; (**b**) Hemolymph of infected mosquitoes were pooled and viral RNA copies were quantified at 24 h.p.i.

Establishment of viral infections of CHIKV, DENV and co-infections of both CHIKV/DENV in mosquitoes were determined at three time points namely, 24 h.p.i., 48 h.p.i. and 72 h.p.i. by quantifying viral RNA copies using qRT-PCR of five individual mosquitoes at all the three time points and also in hemolymph extracted from pool of 50 mosquitoes at 24 h.p.i. (Figure 1). Analysis of individual mosquitoes showed average value of 3.66 × 10^4^, 7.88 × 10^4^ and 6.30 × 10^5^ CHIKV viral RNA copy at 24 h.p.i., 48 h.p.i. and 72 h.p.i. Similarly, average 9.16 × 10^3^, 2.43 × 10^4^ and 2.95 × 10^5^ DENV viral RNA copies were present at 24; 48 and 72 h.p.i. respectively. In case of co-infected samples, viral RNA copies of CHIKV and DENV at all the three time points were in range of 8.73 × 10^3^ to 5.93 × 10^5^ and 2.91 × 10^3^ to 1.82 × 10^6^ respectively. In case of pool samples of hemolymph, quantification of viral RNA copies for both the viruses were performed at 24 h.p.i. and it was found that CHIKV RNA copies were present in the range of 2.15 × 10^3^ to 1.29 × 10^4^ and DENV RNAs were in the range of 9.96 × 10^2^ to 3.29 × 10^3^.

### 3.2. Metabolites Profiling of CHIKV, DENV, and CHIKV/DENV Co-Infected Hemolymph of Aedes Mosquitoes

Post peak acquisition, the 12 samples were divided into their respective groups and were processed further with the help of the MetaboAnalyst tool. A total of 1577 peaks were present, including an average of 131.4 peaks for each sample. For grouping the proximal peaks, a moving window of 0.03 and a step of 0.015 ppm were used and the peaks were aligned to their median positions across the samples. The peaks were summed if more than one peak belonging to the same sample was found in the same group. Fifty-nine peaks present in less than 50% of the samples were excluded from analysis. Phenotype information was not used while filtering the data. Samples median was used to perform row-wise normalization, whereas datasets were log normalized and data scaling was performed using Autoscaling method (Supplementary Figure S2).

Global metabolite profiling of 12 hemolymph samples extracted from control, i.e., PBS-injected mosquitoes (MM), CHIKV mono-infected (CM), DENV mono-infected (DM), and co-infected (CD) *Aedes* mosquitoes revealed seven significant metabolites with *p*-value threshold of 0.13 (Table 1, Supplementary Figure S3).

**Figure 2:**
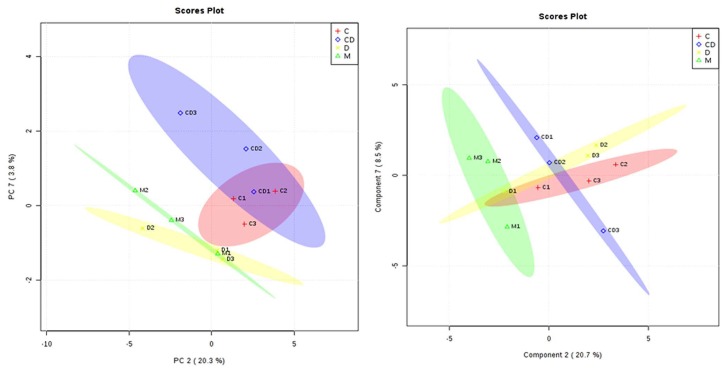
Score plots of PCA analysis and PLS-DA analysis showing best separations between samples.

Furthermore, to check the variance in datasets, we performed PCA and PLS-DA analyses. In PCA analysis, pair-wise score plots of the selected five principal components (PC) showed 40.4% variance in PC 1, 20.3% in PC 2, 11.6% in PC 3, 9.7% in PC 4, and 6.6% in PC 5 (Supplementary Figure S4a). PLS-DA analysis performed on data also showed similar results, with 31.3% variance in component 1, 20.7% in component 2, 14.5% in component 3, 9.8% in component 4, and 5.3% in component 5 (Supplementary Figure S4b). The score plot between two selected PCs and the best components from the PLS-DA analysis showing the best separation between the samples are shown in Figure 2, where the PCA plot is drawn between PC 2 (20.3%) and PC 7 (3.8%) and the PLS-DA plot is shown between component 2 (20.7%) and component 7 (8.5%).

**Figure 3:**
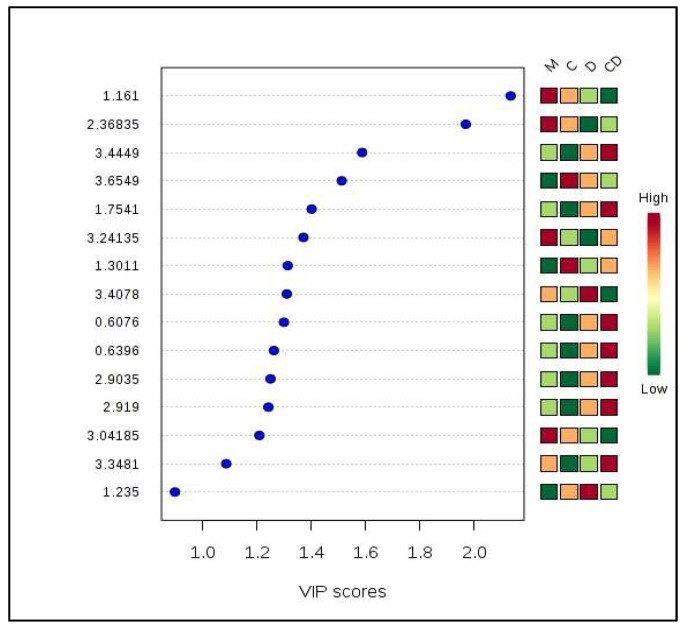
Important metabolites identified by PLS-DA analysis.

On the basis of the relative concentration of the important metabolites identified using variable importance in projection (VIP) scores during PLS-DA analysis, two metabolites (3-hydroxymethylglutaric acid and threitol) were up-regulated in CHIKV-infected samples in comparison to the other mono and co-infected samples (Figure 3). Likewise, four metabolites (isobutyric acid, taurine, cysteine, and ethanol) were down-regulated in the co-infected samples whereas seven metabolites (hypotaurine, asparagine, ethane sulfonic acid, acetylenedicarboxylate, biuret, 1,4-diaminobutane dihydrochloride, and dihysrouracil) showed up-regulation in the co-infected samples. Two metabolites (methylmalonic acid and taurine) were found to be present in significantly up-regulated in DENV-infected mosquitoes (Figure 3).

Pathway analysis of these important metabolites identified taurine and hypotaurine metabolism pathways as being significantly regulated, with a *p*-value of 1.88 × 10^−5^, followed by thiamine metabolism (*p* = 00.063562) (Table 2).

### 3.3. Pair-Wise Metabolites Profiling of CHIKV, DENV, and Co-Infected Hemolymph of Aedes Mosquitoes in Comparison to Control Mosquitoes

**Figure 4:**
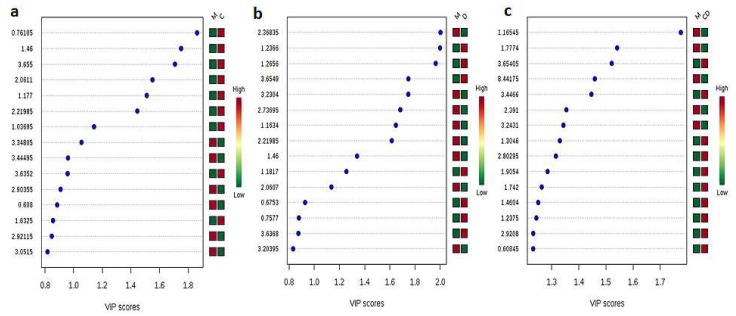
The figure represents important metabolites identified by PLS-DA using VIP score. (**a**) Important metabolites upon CHIKV infection; (**b**) Important metabolites upon DENV infection; (**c**) Important metabolites upon co-infection.

In addition to the global metabolite profiling of hemolymph samples, we also performed pair-wise metabolites profiling of PBS-injected control mosquitoes and other infected mosquito samples. Data filtering and normalization steps were performed using the same strategy explained in global metabolite profiling. CHIKV-infected mosquito samples showed formation of 53 peak groups with an average of 88.8 peaks per sample when compared to controls. PCA analysis showed 56.7% variance in PC 1, 22.7% in PC 2, 10.2% in PC 3, 8.3% in PC 4, and 2.1% in PC 5 (Supplementary Figure S5a). PLS-DA analysis showed 53.6% variance in component 1, 12.8% in component 2, 21.1% in component 3, 10% in component 4, and 2.5% in component 5 (Supplementary Figure S5b). PLS-DA analysis also identified 15 important metabolites using VIP scores; out of these, nine metabolites (aminoadipic acid, erythritol, propionic acid, *N*-acetylglutamic acid, 3-aminoisobutanoic acid, *N*-acetyl-l-cysteine, threitol, l-alanine, and caproic acid) were up-regulated in CHIKV-infected mosquitoes, and six metabolites (asparagine, l-cysteine, biuret, ethane sulfonic acid, hypotaurine, and dihydrouracil) concentrations were down-regulated in the infected mosquitoes in comparison to control samples (Figure 4a). The pathway analysis of these metabolites predicted taurine and hypotaurine metabolism to be the most significant pathways, with a *p*-value of 0.001572 (Table 2).

During PCA analysis, DENV-infected mosquito samples showed 43.1% variance in PC 1, 25.1% in PC 2, 19.3% in PC 3, 3.8% in PC 4, and 5.7% in PC 5 when compared to controls (Supplementary Figure S5c). PLS-DA analysis showed 23.8% variance in the case of component 1, 10% in component 2, 40.3% in component 3, 16.9% in component 4, and 9.1% in component 5 (Supplementary Figure S5d). PLS-DA analysis also identified 15 important metabolites using VIP scores; out of these, seven metabolites (methylmalonic acid, ethane sulfonic acid, threitol, R-pantolactone, acetylenedicarboxylate, caproic acid and erythritol) were present in higher concentrations in DENV-infected mosquitoes, and 8 metabolites (2-aminoethyl dihydrogen phosphate, *N*-acetyl-l-cystine, alanine, *N*-acetylglutamic acid, ethanol, citramalic acid, agmatine sulfate, and isobutyric acid) were down-regulated in DENV-infected mosquitoes in comparison to control samples (Figure 4b). Pathway analysis of these metabolites showed no significant pathway (*p* ≤ 0.05), nevertheless, glycosylphosphatidylinositol-anchor biosynthesis pathway was the topmost predicted pathway with *p* = 0.0939, followed by glycolysis or gluconeogenesis (*p* = 0.20213).

In the case of CHIKV–DENV co-infected mosquitoes, PCA analysis showed 55.8% variance in PC 1, 23.7% in PC 2, 11.9% in PC 3, 4.4% in PC 4, and 4.1% in PC 5 (Supplementary Figure S5e). PLS-DA analysis showed similar results, with 53.6% variance in the case of component 1, 19.3% in component 2, 10.9% in component 3, 11.9% in component 4, and 4.4% in component 5 (Supplementary Figure S5f). PLS-DA analysis also identified 15 important metabolites using VIP scores; out of these, 10 metabolites (methylmalonic acid, threitol, dihydrouracil, 3-hydroxymethylglutaric acid, L-dihydroorotic acid, 4-acetamidobutanoic acid, alanine, asparagine, and biuret) were up-regulated in CHIKV–DENV co-infected mosquitoes and 5 metabolites (glutaric acid, quinolinic acid, succinic acid, taurine, and ethanol) were down-regulated in co-infected mosquitoes when compared to control samples (Figure 4c). Similar to the pathway analysis results of DENV-infected mosquitoes, the pathway analysis of important metabolites of co-infected samples showed no significant pathway (*p* ≤ 0.05) and similar to the global metabolites profiling results and pair-wise analysis of CHIKV-infected samples results, taurine and hypotaurine metabolism were the topmost predicted pathways (*p* = 0.063562), followed by pantothenate and CoA biosynthesis (*p* = 0.12343) (Table 2).

### 3.4. Integration of -Omics Data

#### 3.4.1. Data Selected for Study

Using the search described in the Materials and Methods section, eight studies related to proteomics, ten studies related to transcriptomics, and one study of metabolomics were selected for further analysis. We included our lab-generated data in the study to make the analysis more meaningful. There was no relevant data available related to metabolomics profiling of the hemolymph of mosquito upon CHIKV or DENV infection except for our lab-generated data. Our analysis revealed that in the case of the proteomics study, 2D-DIGE was the preferred technique for studying the effect of infections (Table 3).

#### 3.4.2. Differentially Regulated Transcripts and Pathway Analysis

The literature survey performed on transcriptomics studies done on mosquitoes upon DENV infection or CHIKV infection presented nine datasets related to DENV infection but no study related to CHIKV. Recently published work from our group was the only study found on *Aedes* upon CHIKV, DENV, and co-infection [16].

Transcripts dysregulated upon DENV infection from the nine datasets were divided on the basis of the different tissues, namely, midgut, salivary gland, and carcass. Out of the nine studies, one study was performed on *Ae. albopictus* midgut and carcass. To maintain the homogeneity of analysis, the *Ae. albopictus* IDs were converted to their *Ae. aegypti* homologs. All the significantly differentially regulated transcripts (*p* ≤ 0.05) were collated according to their corresponding tissues. A total of 460 transcripts were differentially regulated in the case of midgut infected with DENV, out of which 77 transcripts were upregulated and rest were downregulated. A total of 311 transcripts were regulated in the salivary gland upon DENV infection, of which 213 transcripts were upregulated and 98 were downregulated. Similarly, 949 significantly transcripts were found in the case of carcass upon DENV infection, with 422 transcripts being upregulated and 527 being downregulated.

The differentially regulated transcripts were further subjected to pathway analysis using KOBAS 3.0 web server and significant pathways with *p* ≤ 0.05 were identified. 19 pathways (metabolic pathways; oxidative phosphorylation; biosynthesis of amino acids; lysosome; citrate cycle (TCA cycle); carbon metabolism; 2-oxocarboxylic acid metabolism; glycosphingolipid biosynthesis-globo series; arginine and proline metabolism; glycine, serine, and threonine metabolism; fatty acid biosynthesis; caffeine metabolism; arginine biosynthesis; one carbon pool by folate; glycosaminoglycan degradation; other glycan degradation; pyruvate metabolism; glycosphingolipid biosynthesis-ganglio series; tryptophan metabolism) were significantly differentially regulated in DENV-infected midgut of mosquito. The pathway analysis of differentially regulated transcripts of the salivary gland showed 11 pathways to be significant, namely, glycine, serine and threonine metabolism; one carbon pool by folate; metabolic pathways; lysosome; biosynthesis of amino acids; nitrogen metabolism; phototransduction—fly; FoxO signaling pathway; arginine and proline metabolism; carbon metabolism; and starch and sucrose metabolism. Similarly, in the case of carcass of DENV-infected mosquito, 16 pathways were significantly regulated, namely, metabolic pathways; oxidative phosphorylation; biosynthesis of amino acids; pyruvate metabolism; carbon metabolism; citrate cycle (TCA cycle); one carbon pool by folate; glycolysis/gluconeogenesis; glycine, serine, and threonine metabolism; lysosome; FoxO signaling pathway; neuroactive ligand–receptor interaction; 2-oxocarboxylic acid metabolism; glyoxylate and dicarboxylate metabolism; fatty acid biosynthesis; and histidine metabolism (Table 4, Table 5 and Table 6).

#### 3.4.3. Differentially Regulated Proteins and Pathway Analysis

**Figure 5:**
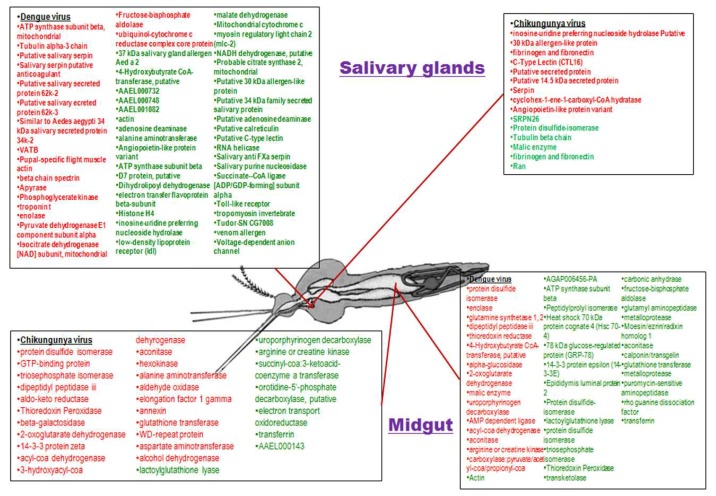
The up regulated (**red fonts**) and down regulated (**green fonts**) proteins of mosquito’s salivary gland and midgut upon chikungunya and dengue infection.

The proteomics data from the selected seven studies were curated and categorized into two main groups, namely, salivary glands and midgut. The midgut of mosquitoes upon DENV infection showed a total of 38 proteins to be dysregulated. Of these, 15 proteins were upregulated and the rest were downregulated. In the case of CHIKV, 29 midgut proteins showed differential regulation, of which 21 proteins were upregulated and 8 proteins were downregulated. A comparative analysis of the regulated midgut proteins of both the viruses showed 12 commonly dysregulated proteins, namely, 2-oxoglutarate dehydrogenase, lactoylglutathione lyase, triosephosphate isomerase, acyl-co-a-dehydrogenase, uroporphyrinogen decarboxylase, glutathione transferase, thioredoxin peroxidase, protein disulfide isomerase, arginine or creatine kinase, dipeptidyl peptidase iii, aconitase, and transferrin (Figure 5). A total of 53 differentially regulated proteins were found in the case of salivary gland upon DENV infection and 15 differentially regulated proteins were found in the case of CHIKV infection (Figure 5). The fewer numbers of proteins detected in the case of CHIKV may be due to the lower number of proteomics studies related to CHIKV and its vector. In the case of DENV-infected salivary gland, out of the 53 dysregulated proteins, 18 proteins were upregulated and 35 were downregulated, and in the case of CHIKV infected salivary gland, nine proteins were upregulated and six proteins were downregulated (Figure 5).

A comparative analysis of the regulated salivary gland proteins upon DENV and CHIKV infection revealed three proteins commonly differentially regulated by both the viruses, namely, inosine-uridine preferring nucleoside hydrolase, angiopoietin-like protein variant, and a putative 30 kDa allergen-like protein.

The differentially regulated proteins of both midgut and salivary gland were further analyzed separately to identify their respective pathways. Pathway analysis of proteins of midgut showed 16 pathways significantly differentially regulated in the case of DENV and 24 pathways were found to be significantly perturbed in the case of CHIKV-infected midgut (Supplementary Figure S6a). Out of these pathways, 10 pathways were common between CHIKV and DENV, namely, alanine, aspartate, and glutamate metabolism; arginine biosynthesis; porphyrin and chlorophyll metabolism; 2-oxocarboxylic acid metabolism; fructose and mannose metabolism; arachidonic acid metabolism; glycolysis/gluconeogenesis; biosynthesis of amino acids; carbon metabolism; and metabolic pathways.

Pathway analysis of dysregulated proteins of the salivary gland showed a total of 16 significantly differentially regulated pathways in the case of DENV and 10 differentially regulated pathways in the case of CHIKV. Three pathways were common between CHIKV and DENV, namely, pyruvate metabolism, carbon metabolism, and propanoate metabolism (Supplementary Figure S6b).

#### 3.4.4. Integration of Pathways to Understand Mechanism of Oxidative Stress

Integrating multi-omics data is still a challenge and there is no generalized algorithm to efficiently integrate the omics data from different fields. Transcriptomics data analysis revealed a total of 460 transcripts differentially regulated in DENV infected midgut and 311 transcripts were differentially regulated in salivary gland upon DENV infection. Data analysis of proteomics data collected from the literature reveals 53 proteins were differentially regulated in DENV-infected salivary gland and 15 proteins in salivary gland infected with CHIKV. In the case of midgut infected with CHIKV and DENV, 38 and 29 proteins, respectively were differentially regulated. The proteomics study performed using whole body of *Ae. aegypti* in our lab (unpublished) showed that 10 proteins in the case of CHIKV-infected mosquito samples, 20 proteins in DENV-infected mosquitoes, and 10 proteins upon CHIKV/DENV co-infection were differentially regulated. Similarly, an in-house transcriptomics profiling of CHIKV/DENV mono- and co-infection of whole mosquitoes, 190 genes upon CHIKV infection, 37 genes upon DENV infection, and 100 genes upon CHIKV/DENV co-infection were found to be differentially expressed. In metabolomics profiling of mono- and co-infected *Aedes* mosquitoes, a total of 29 metabolites were found to be significant.

**Figure 6:**
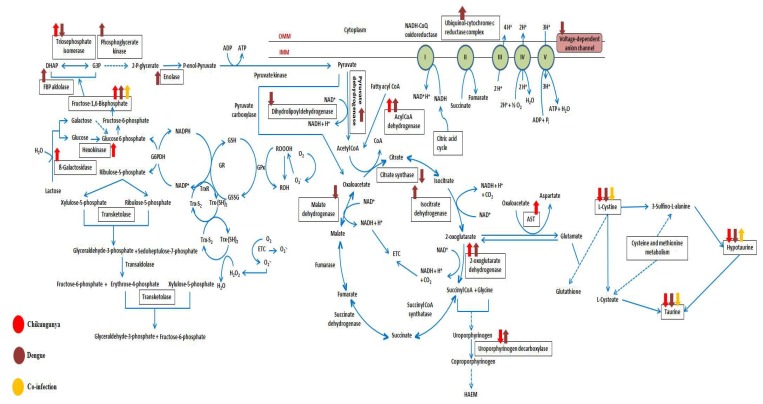
The figure represents important biomolecule predicted from transcriptome, proteome and metabolomics study related to oxidative stress upon CHIKV/DENV mono- and coinfection. The biomolecules related to oxidative stress identified in the present study are written inside the box. Red colour represents proteins from CHIKV infected samples, Brown represents proteins from DENV infected samples and yellow colour represents results obtained from co-infection data.

All these differentially regulated molecules from different studies were classified into different pathways, and the genes, proteins, and metabolites which belong to oxidative stress-related pathways were extracted and an integrated pathway model was generated (Figure 6, Table S1).

Metabolic profiling of mosquitoes suggested taurine and hypotaurine pathways have a role during oxidative stress upon viral infection by affecting glutathione metabolism through l-cysteine. Integration of molecules with each other additionally revealed that other pathways such as glycolysis, pentose phosphate pathway (PPP), citric acid cycle, and glutathione metabolism also played an important role during oxidative stress (Figure 6).

## 4. Discussion

To understand the role of oxidative stress during CHIKV/DENV mono- and co-infection in a more comprehensive manner, we collected different -omics data from the public database, performed metabolomics profiling of mono and co-infected mosquitoes using NMR, analyzed the results and classified them into their respective pathways, and integrated the pathways to generate oxidative stress-related pathway network (Figure 6). Analysis of the data showed that the pathways worked in a harmonized manner to regulate the oxidative stress mechanism upon arboviral infections in *Aedes*.

One of the most significant pathways to be regulated in our study was oxidative phosphorylation. This pathway has been shown to be regulated in other studies involving CHIKV infection in mosquito cells [35]. A transcriptomics study performed upon mono and co-infection of CHIKV and DENV in *Aedes* showed oxidative phosphorylation to be downregulated at early stages of CHIKV infection [16]. It has been proved that early infection of virus modulates bioenergetics of mitochondria to increase the efficiency of viral replication, which could a possible cause for the regulation of this pathway [36]. 

Similarly, PPP produces the ribose needed for the synthesis of nucleic acid which plays an important role during the replication of viruses [37,38]. Fructose-1,6-biphosphatase was found to be differentially regulated in CHIKV and DENV mono-infections as well as co-infections, supporting the use of ribose during viral replication. p17 matrix protein of HIV-1 was found to induce fructose-1,6-bisphosphatase [39]. It has been reported that when glucose decreases in cells, the phosphorylation of fructose-1,6-biphoshphatase can switch glycolysis and gluconeogenesis to compensate for the requirement of energy [40].

Glycolysis/gluconeogenesis pathways were highly utilized by CHIKV and DENV. HCV virus has been shown to increase the production of glucose by promoting gluconeogenesis [41]. Regulation of the glycolysis pathway upon infection with CHIKV, DENV-2, and different pathogens has also been reported [30,36,42,43]. We found glycolysis/gluconeogenesis to play an important role upon arboviral infection.

We also found that citric acid cycle/TCA cycle play a central role upon viral infection and provided the necessary intermediate products to other pathways like fatty acid metabolism. In other studies, citric acid cycle was found to be regulated in the salivary gland of *Aedes* upon DENV-2 infection where citrate synthase, malate dehydrogenase, and succinyl CoA-synthase were down-regulated whereas pyruvate dehydrogenase and isocitrate dehydrogenase were upregulated [33]. Enzyme activity of the pathway was shown to be increased in influenza virus-infected MDCK cells [44].

Fatty acyl CoA was another molecule that was regulated in our study. Fatty acid metabolism, which includes fatty acid biosynthesis and beta-oxidation, has been reported to be differentially regulated upon arboviral infection in the case of both mammals and mosquitoes [45,46]. Sphingolipids and cholesterol are also required by CHIKV during entry and membrane fusion into the cell [47]. Lipid metabolism has also been up-regulated in the midgut upon CHIKV and DENV infection [30]. The production of influenza A virus decreases when fatty acid synthesis is inhibited [48].

Carbon metabolism forms metabolic precursors via several enzymatic steps [49]. The major carbon utilization pathways are glycolysis, PPP, citrate cycle, and methane metabolism. The role of carbon metabolism and its association with virulence has been extensively studied in pathogens like *Listeria monocytogenes*, *Salmonella enteric* subsp., *Shigella flexneri*, and *Mycobacterium tuberculosis* [50,51,52,53] but nothing much is known about the role of the pathway in vectors.

Taurine and hypotaurine metabolism also emerged as important pathways in the metabolomics study. l-Cysteine, taurine, *N*-acetyl-l-cysteine, and hypotaurine were found to be important and regulated during infection. l-Cysteine is a connecting link between approximately 15 metabolic pathways. It is known to be a precursor of the well-known antioxidant glutathione. It is also important for production of collagen. The role of l-cysteine in infection has not been studied. On the other hand, the role of *N*-acetyl-l-cysteine during infections is established. In 2010, Geiler et al. have shown the importance of this compound in inhibiting the replication of H5N1 influenza A virus and it was found that it also inhibits the production of viral-induced pro-inflammatory molecules [54]. Similarly, another study have shown the role of *N*-acetylcysteine in the treatment of *Helicobacter pylori* [55]. Taurine is found to be an important component in brain, muscle tissue, and retina and it is also found to be important for many functions in the central nervous system and has been shown to reduce infection-associated effects during preterm labor in animal models [56]. Taurine has been shown to play an important role during oxidative stress as it inhibits the production of reactive oxygen species (ROS) and maintains the integrity of mitochondria by controlling the electron transport chain. Although this mechanism was discovered in vertebrates, the role it plays during oxidative stress may be also be important during pathogen infection [57].

The equilibrium state of redox is shown to play an important role in physiological activities of cells and also on pathogen infection [58]. High levels of oxidative stress has been shown to interfere with pathogen infection [59,60]. Oxidative stress has been shown to increase in cell lines of lepidopteran origin upon nucleo-polyhedroviral infection [61]. In mosquitoes, oxidative stress is altered during blood meal as heme is released upon digestion of blood and induces oxidative stress in midgut by reacting with other ROS factors which may prove fatal for the insects [62,63]. In case of *Ae. aegypti* it has been shown that the mosquitoes inhibit production of ROS by inhibiting the activity of dual oxidase [64]. Reports have emphasized the importance of utilizing a integromics approach to understand the various mechanisms of pathogenesis [65]. Through our present study, we have deciphered the network of oxidative stress during arboviral infections in *Aedes*.

## Figures and Tables

**Table 1:** Important features identified by one-way ANOVA and post-hoc analysis.

S. No.	Peaks (ppm)	Chi-Squared	*p*-Value	−log 10(*p*)	FDR
1	3.697	6.69	0.08	1.08	0.65
2	3.655	6.59	0.09	1.06	0.65
3	1.161	6.49	0.09	1.04	0.65
4	1.635	6.23	0.1	1	0.65
5	2.061	6.08	0.11	0.97	0.65
6	1.713	5.82	0.12	0.92	0.65
7	2.368	5.62	0.13	0.88	0.65

**Table 2:** Significant pathways identified upon CHIKV, DENV, and co-infection.

Sample	Pathway Name	Total	Hits	*p*-Value	Compounds
Global metabolite profiling	Taurine and hypotaurine metabolism	6	3	1.88 × 10^−5^	l-Cysteine, taurine, hypotaurine
	Thiamine metabolism	6	1	0.063562	l-Cysteine
CHIKV (CM)	Taurine and hypotaurine metabolism	6	2	0.0015716	l-Cysteine, hypotaurine
	Thiamine metabolism	6	1	0.063562	l-Cysteine
DENV (DM)	Glycosylphosphatidylinositol-anchor biosynthesis	11	1	0.093946	Ethanol
	Glycolysis or Gluconeogenesis	25	1	0.20213	Ethanol
Co-infection (CD)	Taurine and hypotaurine metabolism	6	1	0.063562	Taurine
	Pantothenate and CoA biosynthesis	12	1	0.12343	Dihydrouracil

**Table 3:** List of selected data used in this study.

S. No.	Organism	Body Part/Source	Technique	Virus(es)	References
Transcriptomics					
1	*Aedes aegypti*	Midgut	cDNA Microarray	DENV2	[20]
2	*Aedes aegypti*	Salivary gland	DGE	DENV2	[21]
3	*Aedes aegypti*	Whole mosquito	Microarray	DENV2	[22]
4	*Aedes albopictus*	Midgut	RNA-seq	DENV2	[23]
		Carcass	RNA-seq		
5	*Aedes aegypti*	Salivary gland	Microarray	DENV2	[24]
		Chemosensory apparatus			
6	*Aedes aegypti*	Midgut	Microarray	DENV2	[25]
		Carcass			
7	*Aedes aegypti*	Midgut	RNA-seq	DENV2	[26]
		Salivary gland			
		Carcass			
8	*Aedes aegypti*	Aag2 cells	Microarray	DENV2	[27]
9	*Aedes aegypti*	Whole mosquito	Microarray	DENV2	[28]
10	*Aedes aegypti*	Whole mosquito	RNA-seq	CHIKV and DENV2	[16]
Proteomics					
1	*Aedes albopictus*	Salivary gland	2D-DIGE; MALDI TOF/TOF	DENV2	[29]
		Midgut		DENV2	
		C6/36		DENV2	
2	*Aedes aegypti*	Midgut	2D-DIGE	CHIKV and DENV2 infection	[30]
3	*Aedes aegypti*	Salivary gland	2D-DIGE; MALDI TOF/TOF	CHIKV	[31]
4	*Aedes aegypti*	Saliva	2-D; Nano LC-MS/MS	DENV2	[32]
5	*Aedes aegypti*	Salivary glands	2-D; Nano LC-MS/MS	DENV2	[33]
6	*Aedes albopictus*	C6/36	2D-DIGE	DENV1; DENV3	[34]
7	*Aedes albopictus*	C6/36	2D-PAGE; Maldi TOF/TOF	CHIKV	[35]
8	*Aedes aegypti*	Whole body	LC-MS/MS	CHIKV and DENV2	Data link: 10.6084/m9.figshare.5746134
Metabolomics					
1	*Aedes aegypti*	Hemolymph	NMR	CHIKV and DENV2	This study

**Table 4:** Significant pathways of differentially regulated transcripts of midgut.

Pathways	Input Number	Total Number of Transcripts/Genes	*p*-Value
Metabolic pathways	85	929	56 × 10^−21^
Oxidative phosphorylation	34	142	1.36 × 10^−19^
Biosynthesis of amino acids	12	57	3.14 × 10^−7^
Lysosome	12	81	8.21 × 10^−6^
Citrate cycle (TCA cycle)	8	34	1.50 × 10^−5^
Carbon metabolism	11	98	0.00019
2-Oxocarboxylic acid metabolism	4	16	0.001865
Glycosphingolipid biosynthesis—globo series	3	9	0.003683
Arginine and proline metabolism	5	37	0.005454
Glycine, serine, and threonine metabolism	5	38	0.006036
Fatty acid biosynthesis	3	12	0.007168
Caffeine metabolism	3	13	0.008645
Arginine biosynthesis	3	14	0.010289
One carbon pool by folate	3	14	0.010289
Glycosaminoglycan degradation	3	15	0.0121
Other glycan degradation	3	19	0.021077
Pyruvate metabolism	4	37	0.024909
Glycosphingolipid biosynthesis—ganglio series	2	8	0.028854
Tryptophan metabolism	3	26	0.043514

**Table 5:** Significant pathways of differentially regulated transcripts of salivary gland.

Pathways	Input Number	Total Number of Transcripts/Genes	*p*-Value
Glycine, serine, and threonine metabolism	5	38	0.001091
One carbon pool by folate	3	14	0.00343
Metabolic pathways	27	929	0.017225
Lysosome	5	81	0.020921
Biosynthesis of amino acids	4	57	0.025549
Nitrogen metabolism	2	13	0.030062
Phototransduction—fly	3	35	0.032108
FoxO signaling pathway	4	62	0.032892
Arginine and proline metabolism	3	37	0.03662
Carbon metabolism	5	98	0.041077
Starch and sucrose metabolism	3	40	0.043962

**Table 6:** Significant pathways of differentially regulated transcripts of carcass.

Pathways	Input Number	Total Number of Transcripts/Genes	*p*-Value
Metabolic pathways	111	929	2.84 × 10^−12^
Oxidative phosphorylation	34	142	7.48× 10^−11^
Biosynthesis of amino acids	15	57	72 × 10^−6^
Pyruvate metabolism	11	37	3.94× 10^−5^
Carbon metabolism	18	98	3 × 10^−5^
Citrate cycle (TCA cycle)	10	34	9.52 × 10^−5^
One carbon pool by folate	6	14	0.000499
Glycolysis/gluconeogenesis	9	44	0.001988
Glycine, serine, and threonine metabolism	8	38	0.00299
Lysosome	12	81	0.004325
FoxO signaling pathway	10	62	0.00524
Neuroactive ligand–receptor interaction	8	51	0.013724
2-Oxocarboxylic acid metabolism	4	16	0.020663
Glyoxylate and dicarboxylate metabolism	5	30	0.038834
Fatty acid biosynthesis	3	12	0.04422
Histidine metabolism	3	12	0.04422

## Supplementary Materials

The following are available online at http://www.mdpi.com/1999-4915/10/6/314/s1, Figure S1. Workflow followed in the study; Figure S2. Intensity of metabolites before and after normalization; Figure S3. Graphical representation of ANOVA results; Figure S4. Pair-wise score plots showing the variance: (a) PCA analysis and (b) PLS-DA analysis; Figure S5. PCA (a), (c), (e) and PLS-DA (b), (d), (f) plots pair-wise analysis of CHIKV, DENV, and co-infected samples, respectively; Figure S6. Significantly regulated pathways of proteomics data upon CHIKV and DENV infection in (a) midgut and (b) salivary gland of mosquito. Table S1. List of all genes, proteins and metabolites from this study related to oxidative stress pathways.
