# Supplementary figures and images for: Understanding Oxidative Stress in Aedes during Chikungunya and Dengue Virus Infections Using Integromics Analysis
Source: Viruses. 2018 Jun 9;10(6):314. doi: 10.3390/v10060314 (PMC6024870; doi:10.3390/v10060314)

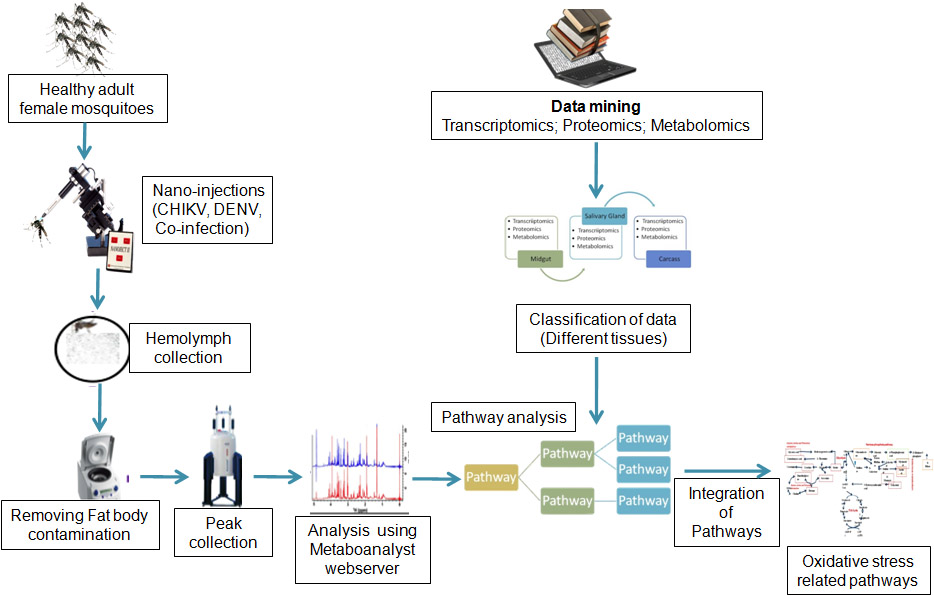

Supplement: Supplementary file 1 [file viruses-10-00314-s001.zip › Supl_material/FIgure S1.jpg]

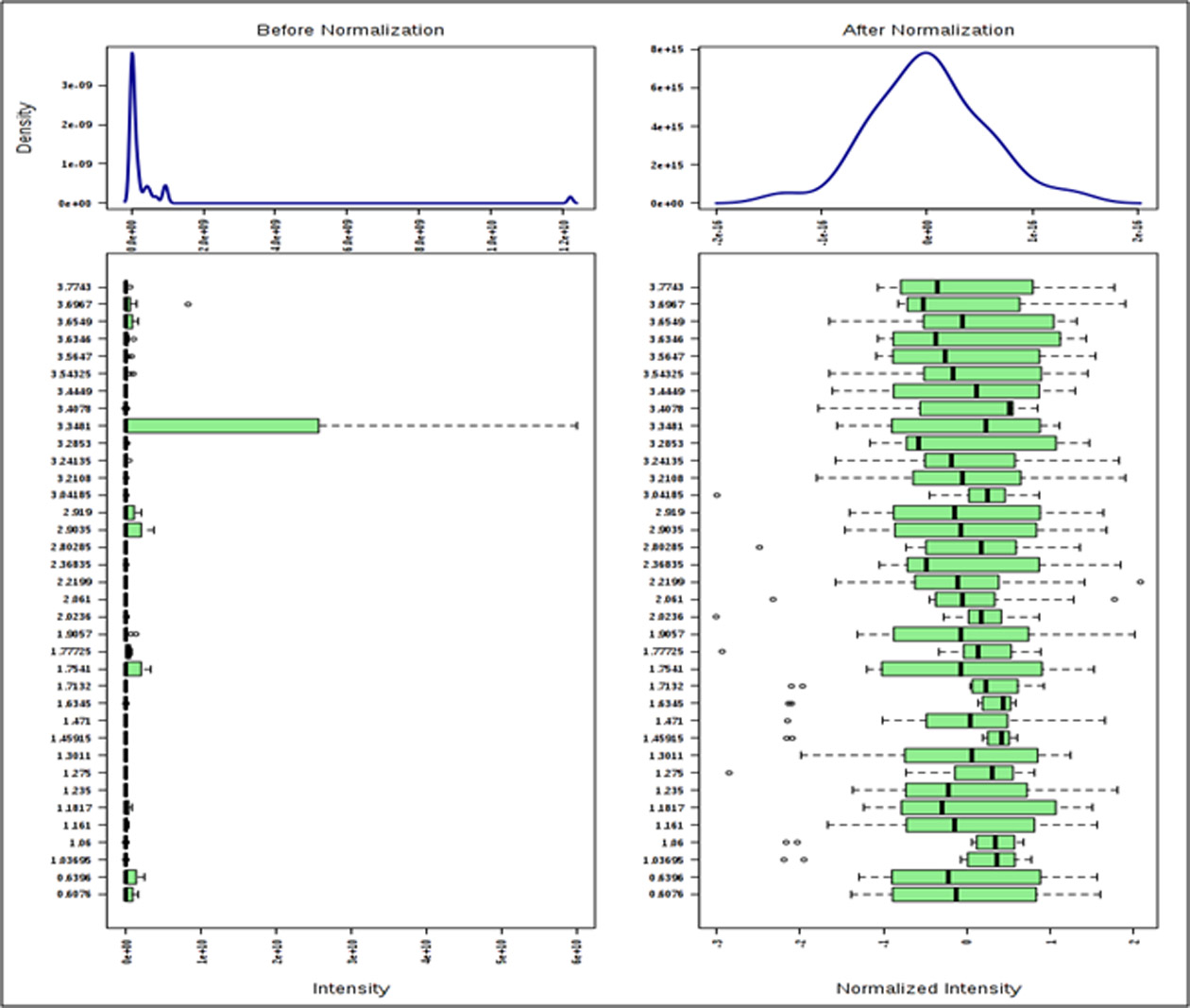

Supplement: Supplementary file 1 [file viruses-10-00314-s001.zip › Supl_material/Figure S2.jpg]

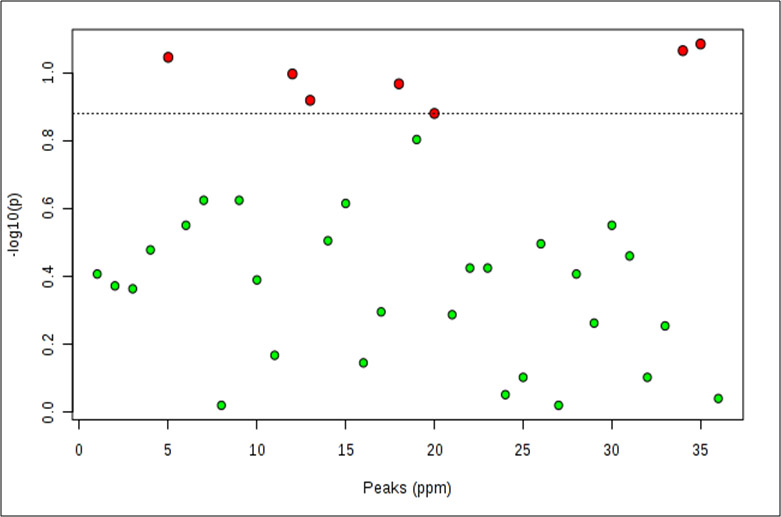

Supplement: Supplementary file 1 [file viruses-10-00314-s001.zip › Supl_material/Figure S3.jpg]

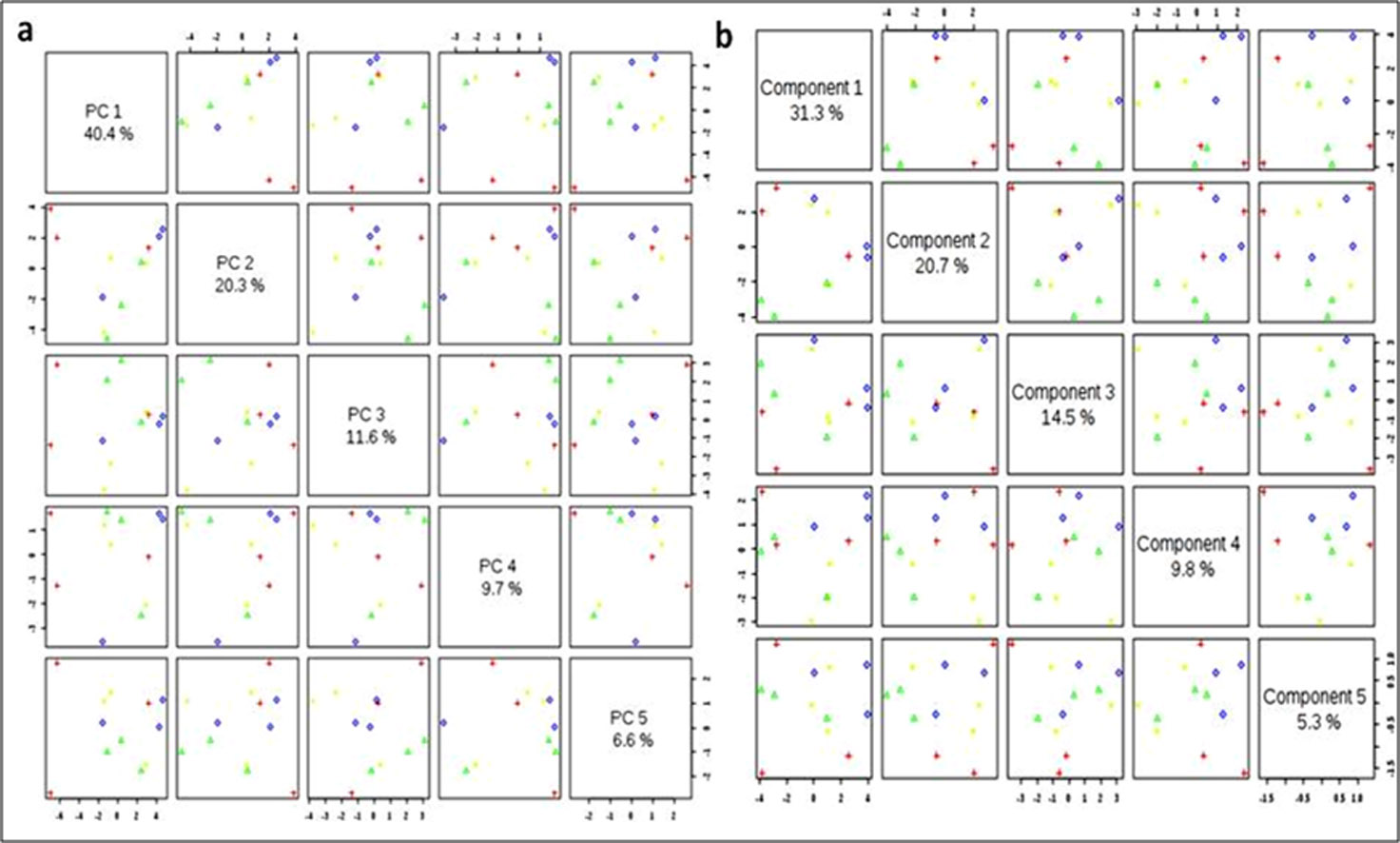

Supplement: Supplementary file 1 [file viruses-10-00314-s001.zip › Supl_material/Figure S4.jpg]

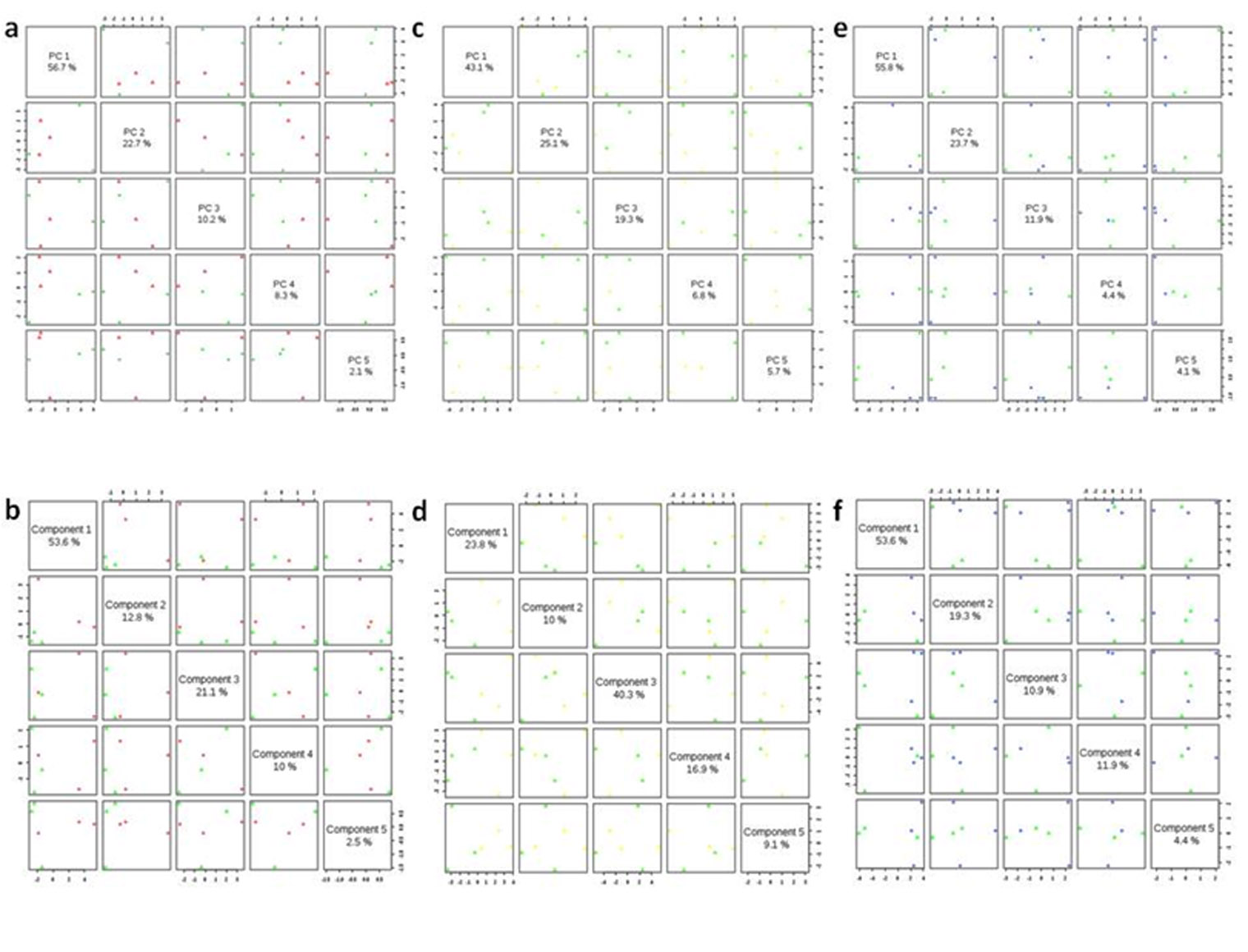

Supplement: Supplementary file 1 [file viruses-10-00314-s001.zip › Supl_material/Figure S5.jpg]

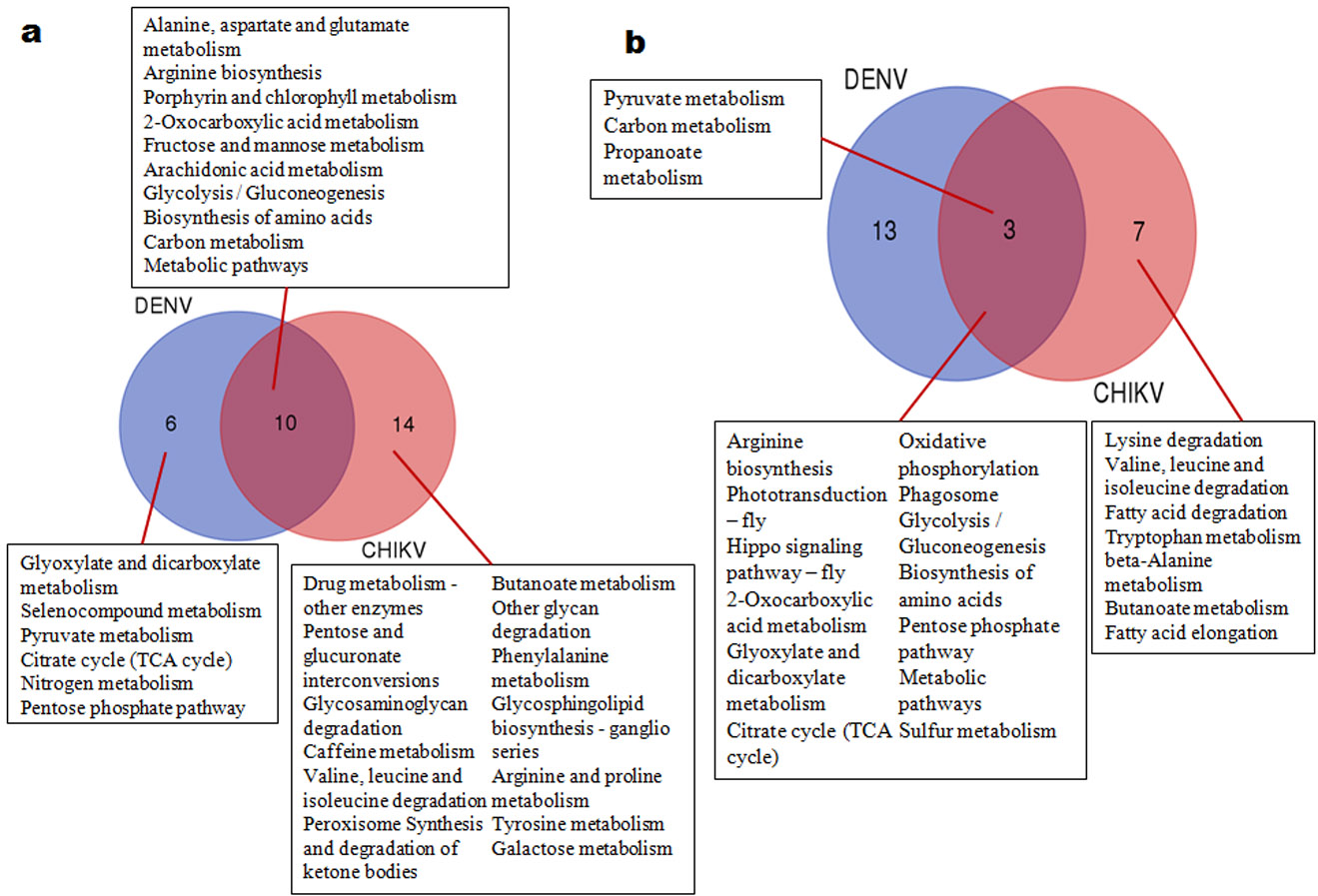

Supplement: Supplementary file 1 [file viruses-10-00314-s001.zip › Supl_material/Figure S6.jpg]
